# Peracetic acid as a disinfectant for wastewater reuse — Regulation (EU) 2020/741 application on a pilot-scale

**DOI:** 10.1007/s10661-023-11313-7

**Published:** 2023-05-20

**Authors:** Rita Dias, Diogo Sousa, Rita Lourinho, Rita Maurício

**Affiliations:** 1 CENSE – Center for Environmental and Sustainability Research & CHANGE - Global Change and Sustainability Institute, NOVA School of Science and Technology, NOVA University Lisbon, Campus de Caparica, 2829-516 Caparica, Portugal; 2 Águas do Tejo Atlântico, AdP—Grupo Águas de Portugal, Lisbon, Portugal

**Keywords:** Peracetic acid, Wastewater reuse, Agricultural irrigation, Disinfection alternative, Operational parameters and implementation, Sustainable development goal 6

## Abstract

Water scarcity affects already a large part of the world's population. To overcome this situation, water management is needed, and wastewater reuse must be implemented and included as a new approach. To achieve that objective water quality must comply with the parameters established in the Regulation (EU) 2020/741 of the European Parliament and the Council of the European Union and new treatment solutions have to be developed. The main goal of this pilot study was to evaluate the peracetic acid (PAA) disinfection efficiency in a real wastewater treatment plant (WWTP) in order to accomplish the wastewater reuse objective. To this end, six disinfection conditions were studied, three PAA doses (5, 10, and 15) and three contact times (5, 10, and 15) based on the commonly used disinfection operational conditions in real WWTP. Comparing the Total Suspended Solids (TSS), turbidity, Biological Oxygen Demand (BOD5) and Escherichia coli content, after and before the disinfection step, was possible to conclude that PAA ensures the Regulation (EU) 2020/741 requirements and that the disinfected effluent can be reused for several uses. All the conditions in which the PAA dose was 15 mg/L and the condition with 10 mg/L of PAA with a contact time of 15 min were the most promising, presenting the second highest water quality class achieved. The results of this study illustrate the potential of PAA as an alternative disinfectant for wastewater treatment and, bring it closer to the water reuse objective by presenting several possibilities for water uses.

## Introduction

Water is an essential resource for living beings, however, there is currently, in some locations, a shortage of this resource (Becerra-Castro et al., 2015; Ofori et al., 2021). According to estimates, in a few decades around 40% of the world's population will experience water stress or scarcity. To overcome this scenario, a new approach in water management is necessary, which must include the reuse of wastewater (Becerra-Castro et al., 2015; Shrivastava et al., 2022).

The reuse of wastewater plays an important role in this new approach, as proven by the sixth goal (clean water and sanitation) of the United Nations Sustainable Development Goals, which has already wastewater reuse as a target (UN, 2016). In addition to the freshwater consumption reduction, the reuse of wastewater also decreases the water bodies' pollution through the reduction of the effluents discharged into aquatic ecosystems (Becerra-Castro et al., 2015), lining up with the circular economy proposals (Ruiz-Rosa et al., 2020). Wastewater can be reused for irrigation (Becerra-Castro et al., 2015; Ofori et al., 2021) and industry and urban uses (Shakeri et al., 2021). However, wastewater reuse carries risks for public health and for the environment that should consider due to the presence of pathogenic microorganisms, disinfection by-products (DBP) and compounds of emerging concern (Rebelo et al., 2020). In this context, the European Union (EU) through the Regulation (EU) 2020/741 of the European Parliament and the Council of the European Union (2020) establish the minimum requirements for water reuse. This regulation that will be applied in 2023 aims to create legislation, monitoring requirements, risk management provisions and transparency for all the EU countries (Shrivastava et al., 2022). This regulation establishes four classes of uses, based on water quality requirements for *Escherichia coli*, Biological Oxygen Demand (BOD_5_), Total Suspended Solids (TSS) and Turbidity (Table 1). When the reuse is for irrigation of pastures or forage the intestinal nematodes content need to be lower than 1 egg/L. This regulation also establishes the limit of *Legionella spp*. content where there is a risk of aerosolization and intestinal nematodes content for irrigation of pastures or forage.

**Table 1:** Classes of water quality for irrigation (Adapted from Regulation (EU) 2020/741)

**Water Quality Class**	**Quality requirements**			
	***E. coli***	**BOD**_**5**_	**TSS**	**Turbidity**
	**(number/100 mL)**	**(mg/L)**	**(mg/L)**	**(NTU)**
**Class A**: All food crops consumed raw where the edible part is in direct contact with reclaimed water and root crops consumed raw.	≤ 10	≤ 10	≤ 10	≤ 5
**Class B**: Food crops consumed raw where the edible part is produced above ground and is not in direct contact with reclaimed water, processed food crops and non-food crops including crops used to feed milk- or meat-producing animals.	≤ 100	≤ 25	≤ 35	-
**Class C**: Food crops consumed raw where the edible part is produced above ground and is not in direct contact with reclaimed water, processed food crops and non-food crops including crops used to feed milk- or meat-producing animals.	≤ 1 000	≤ 25	≤ 35	-
**Class D**: Industrial, energy and seeded crops.	≤ 10 000	≤ 25	≤ 35	-

Despite these limits, the regulation defined that for wastewater reuse is essential secondary treatment and advanced treatment, namely wastewater disinfection. The disinfection process is essential to wastewater reuse (de Oliveira Freitas et al., 2021) since this process leads to the inactivation of the pathogenic microorganism which reduces the public health risk (Luna-Pabello et al., 2009; Ofori et al., 2021).

PAA is an organic peroxyacid or peracid, standing out as one of the most relevant peracids due to its high oxidation potential (da Silva et al., 2020), larger than chlorine or chlorine dioxide oxidation potential (Kitis, 2004). PAA is a wide-spectrum disinfectant (da Silva et al., 2020; Pileggi et al., 2022) with efficacy against bacteria (e.g. faecal coliforms, *E. coli*, *Pseudomonas spp.*, and *Salmonella spp*.) and viruses (Cavallini et al., 2013; da Silva et al., 2020) In the disinfection process PAA breaks down into hydrogen peroxide, oxygen, water, and acetic acid (de Oliveira Freitas et al., 2021), with much less DBP than chlorine (Dong et al., 2022). As a result, PAA is classified as a safe and environmentally friendly disinfectant (de Oliveira Freitas et al., 2021). Besides the above mention characteristics, other advantages of using PAA as a disinfectant are pointed out, such as the low pH dependence, easy implementation (Ao et al., 2021), stability, long lead-time, disinfection residual (storage advantages), low corrosivity, widely available and reasonable cost (Chen & Pavlostathis, 2019). Studies on PAA applicability in wastewater have already been performed. de Oliveira Freitas et al. (2021) compared PAA disinfection performance versus the commonly used chlorine. They concluded that PAA had a better performance than chlorine in the removal of total coliforms (TC) and *E. coli*. and the best performance was obtained with 15 mg/L of PAA and 15 min of contact time. Cavallini et al. (2013) study corroborated that PAA dose up to 10 mg/L can be used as a wastewater disinfectant without significant changes in the effluent’s characteristics. In addition to its use as a disinfectant, recent studies show promising results regarding the use of PAA in the degradation of micropollutants present in treated effluents (Maurício et al., 2020).

Although there are studies about the use of PAA as a wastewater disinfectant, the vast majority of these were carried out on a laboratory scale, and that constitutes a lack of real data. Therefore, there is a knowledge gap in the application of PAA at the industrial scale. The main goal of this work was to evaluate in a pilot-scale, for six months, the PAA disinfection efficiency in a real secondary effluent in Portuguese WWTP (Lisbon area) with wastewater reuse intentions. This goal was also extended to assess its final quality according to the EU's newest legislation in wastewater reuse, the Regulation (EU) 2020/741.

## Materials and methods

### Selection of WWTP

The pilot-scale installation was set in a typical conventional large-scale municipal WWTP located in the Lisbon area (Portugal). This WWTP has a flow higher than 50 000 m^3^/day (more than 200 000 equivalent inhabitants) and includes preliminary, primary, and secondary treatment with conventional activated sludge and nitrogen removal.

### PAA disinfection study

This study was performed for six months, from April to September, in which the PAA (OxyPure^®^ BIO—a mixture of 15% PAA, 24% H_2_O_2_ and 16% acetic acid – Evonik Operations GmbH) was dosed in a 5 m^3^ cylindric tank, equipped with a vertical mixer (TIMSA, model TA) 0.75 kW electric motor and vertical coaxial reducer (600 mm diameter and 100 rpm velocity) located after the secondary clarifiers. In this study six disinfection conditions were tested: 15 mg/L with 15, 10 and 5 min of contact time, henceforth named C15T15, C15T10 and C15T10, respectively; 10 mg/L with 15 and 10 min of contact time, hereafter named C10T15 and C10T10 respectively; and, 5 mg/L and 10 min of contact time, henceforth named C5T10. Each condition was tested for five days in which the water quality parameters were analysed (n = 3).

### Water quality parameters

The water quality parameters were analysed before and after the disinfection with PAA. The determination of BOD_5_ and TSS were performed using the methods described in APHA (1999). *E. coli* were determined according to the methods described in the ISO 9308-2:2012 (International Organization for Standardization, 2012). The turbidity of the samples was measured using the nephelometric method, using a portable turbidimeter 0-1000 NTU HANNA Fast Tracker HI98703.

### Statistical analysis

Statistical analysis was used to identify differences between TSS and turbidity results before and after the disinfection for each condition. This analysis was performed using the Microsoft Excel one-way ANOVA with a significance level of 5%.

## Results and discussion

Wastewater class before and after disinfection with PAA.

Table 2 shows the classes of water use obtained for each water quality parameter in each condition before the disinfection with PAA.

**Table 2:** Class of water use for each condition before disinfection ($\overline{X }\pm \sigma ;n$)

**Condition**	**E. coli** **(MPN/100 mL)**	**BOD**_**5**_ **(mg/L)**	**TSS** **(mg/L)**	**Turbidity** **(NTU)**	**Water quality class**
C15T15	40 000 ± 33 071^a^	6.0 ± 0.0^a^	9.0 ± 4.5^b^	1.7 ± 0.8^a^	Not reusable
C15T10	15 840 ± 4 975^a^	6.0 ± 0.0^a^	4.6 ± 1.3^a^	1.4 ± 0.4^a^	Not reusable
C15T5	27 911 ± 18 202^b^	6.0 ± 0.0^a^	3.8 ± 2.0^b^	1.2 ± 0.3^a^	Not reusable
C10T15	45 133 ± 17 484^a^	6.3 ± 1.4^a^	6.9 ± 7.7^a^	1.2 ± 0.3^a^	Not reusable
C10T10	44 667 ± 12 280^a^	6.9 ± 1.8^a^	4.7 ± 1.2^a^	1.2 ± 0.2^a^	Not reusable
C5T10	12 400 ± 9 655^a^	6.0 ± 0.0^a^	4.4 ± 2.6^a^	1.4 ± 0.6^a^	Not reusable

C – PAA concentration and T – contact time (e.g. C15T15 – means 15 mg PAA/L and 15 min of contact time)

^a^ $$\overline X\pm\sigma;$$ n = 15

^b^ $$\overline X\pm\sigma;$$ n = 9

Based on the obtained results and according to the EU standards, for all the conditions, the wastewater before the disinfection could not be reused.

The water quality parameters after the disinfection and the water quality class achieved for each condition are shown in Table 3.

**Table 3:** Class of water use for each condition after disinfection ($\overline{X }\pm \sigma ;n$)

**Condition**	**E. coli** **(MPN/100 mL)**	**BOD**_**5**_ **(mg/L)**	**TSS** **(mg/L)**	**Turbidity** **(NTU)**	**Water quality class**
C15T15	30 ± 25^a^	15.0 ± 4.8^a^	9.9 ± 6.6^c^	2.4 ± 1.1^a^	B
C15T10	11 ± 6^a^	13.4 ± 3.8^a^	6.2 ± 3.4^a^	1.9 ± 0.8^a^	B
C15T5	97 ± 94^c^	20.4 ± 10.4^c^	6.7 ± 3.6^c^	1.3 ± 0.2^c^	B
C10T15	67 ± 34^a^	12.2 ± 5.2^a^	5.0 ± 1.3^a^	1.4 ± 0.4^a^	B
C10T10	121 ± 69^a^	17.9 ± 7.0^a^	5.2 ± 1.1^a^	1.7 ± 0.3^a^	C
C5T10	6 244 ± 5 160^a^	8.6 ± 5.7^a^	5.8 ± 1.7^a^	1.6 ± 0.7^b^	D

C – PAA concentration and T – contact time (e.g. C15T15 – means 15 mg PAA/L and 15 min of contact time)

^a^ $$\overline{{\varvec{X}}}\pm{\varvec{\sigma} }$$; n = 15

^b^ $$\overline{{\varvec{X}}}\pm{\varvec{\sigma} }$$; n = 14

^c^ $$\overline{{\varvec{X}}}\pm{\varvec{\sigma} }$$; n = 9

^d^ $$\overline{{\varvec{X}}}\pm{\varvec{\sigma} }$$; n = 8

No significant differences were identified between TSS and turbidity before and after disinfection in any of the studied conditions (one-way ANOVA, p > 0.05). Therefore, is possible to conclude that PAA does not interfere with these two parameters. However, when comparing the BOD_5_ results before and after disinfection, PAA led to the BOD_5_ increasing. Such conclusion was also obtained by Wagner et al. (2002) and Luna-Pabello et al. (2009). According to Luna-Pabello et al. (2009) this increase is due to the organic compound component of PAA. However, the BOD_5_ increasing after the disinfection does not affect the possibility of its reuse since the results obtained in all the studied conditions were lower than 25 mg/L.

The results showed a high E. coli content in the wastewater before disinfection in all the conditions. Even though, after disinfection all the studied conditions ensured the quality requirements for wastewater reuse. There were differences in the water quality classes achieved. All the conditions in which the PAA dose was 15 mg/L and the condition C10T15 achieved the class B of the Regulation (EU) 2020/741, while C10T10 and C5T10 achieved class C and D, respectively.

The results obtained in this study are not entirely comparable to the published literature on this subject since the majority do not include all Regulation (EU) 2020/741 parameters. Since the regulation is only applied in the EU, it may explain the lack of studies covering all the parameters required. However, focusing only on the E. coli parameter, de Oliveira Freitas et al. (2021) studied the disinfection with PAA using only the condition C15T15 in the effluent of two different biological treatments. The results were similar (class B water quality), specifically when the effluent was from different treatment operational conditions (anaerobic, aerobic, and anoxic). Although, when used the up flow anaerobic sludge blanket effluent, the disinfected effluent did not achieve the quality requirements that enable wastewater reuse. Pileggi et al. (2022) studied the disinfection of an effluent from the secondary treatment using 4 mg/L of PAA, and 35 min of contact time. The E. coli content achieved was also correspondent to a class B water quality, however, the contact time was much higher. Cavallini et al. (2013) also studied the disinfection of effluent from the secondary treatment using 20 min of contact time and six PAA doses were tested (5, 10, 15, 20, 25, and 30 mg/L). The results reported that only with PAA doses higher than 5 mg/L it can be achieved class C water quality considering E. coli content.

## Conclusion and future developments

The results suggest that PAA can be used as a wastewater disinfectant, ensuring the legal requirements established by the Regulation (EU) 2020/741 to reuse wastewater for irrigation. However, only the conditions in which 15 mg/L was used and the condition C10T15, achieved the second highest water quality class (class B). Among the analysed parameters in this campaign, PAA does not interfere with TSS neither with turbidity. Although PAA leads to an increase in the BOD_5_ value, this increment does not preclude the possibility of reuse. Comparing this study with the results reported in the literature was possible to conclude that despite off PAA ensures the disinfection required to reuse wastewater for irrigation. Further studies in real WWTP should focus on the following aspects: i) the optimization of the PAA dose and the contact time in other to achieve the highest class of water quality of the Regulation (EU) 2020/741; ii) the influence of the wastewater physical–chemical properties on the PAA efficiency, iii) the toxicity of this alternative disinfectant; and iv) its application on different wastewater treatment fields, such as the removal of compounds of emerging concern.

## Data Availability

The data presented in this study are available on request from the corresponding author.
